# Assessment of *ERBB2*/*HER2* Status in *HER2*-Equivocal Breast Cancers by FISH and 2013/2014 ASCO-CAP Guidelines

**DOI:** 10.1001/jamaoncol.2018.6012

**Published:** 2018-12-06

**Authors:** Michael F. Press, Jose A. Seoane, Christina Curtis, Emmanuel Quinaux, Roberta Guzman, Guido Sauter, Wolfgang Eiermann, John R. Mackey, Nicholas Robert, Tadeusz Pienkowski, John Crown, Miguel Martin, Vicente Valero, Valerie Bee, Yanling Ma, Ivonne Villalobos, Dennis J. Slamon

**Affiliations:** 1 Norris Comprehensive Cancer Center, University of Southern California, Los Angeles; 2 Departments of Medicine & Genetics, Stanford University, Stanford, California; 3 International Drug Development Institute, Louvain-la-Neuve, Belgium; 4 University of Hamburg, Hamburg, Germany; 5 Frauenklinik vom Roten Kreuz, Munich, Germany; 6 Department of Oncology, University of Alberta, Edmonton, Canada; 7 Virginia Cancer Specialists/US Oncology Research Network, Fairfax, Virginia; 8 Postgraduate Medical Education Center, Warsaw, Poland; 9 Irish Cooperative Oncology Research Group, St Vincent’s University Hospital, Dublin, Ireland; 10 Instituto de Investigación Sanitaria Gregorio Marañón, CIBERONC, GEICAM, Universidad Complutense, Madrid, Spain; 11 The University of Texas, M.D. Anderson Cancer Center, Houston, Texas; 12 Cancer International Research Group/Translational Research in Oncology, Paris, France; 13 Department of Medicine, Geffen School of Medicine at University of California Los Angeles, Los Angeles

## Abstract

**Importance:**

The 2013/2014 American Society of Clinical Oncology and College of American Pathologists (ASCO-CAP) guidelines for *HER2* testing by fluorescence in situ hybridization (FISH) designated an “equivocal” category (average *HER2* copies per tumor cell ≥4-6 with *HER2*/CEP17 ratio <2.0) to be resolved as negative or positive by assessments with alternative control probes. Approximately 4% to 12% of all invasive breast cancers are characterized as *HER2*-equivocal based on FISH.

**Objective:**

To evaluate the following hypotheses: (1) genetic loci used as alternative controls are heterozygously deleted in a substantial proportion of breast cancers; (2) use of these loci for assessment of *HER2* by FISH leads to false-positive assessments; and (3) these *HER2* false-positive breast cancer patients have outcomes that do not differ from clinical outcomes for patients with *HER2*-negative breast cancer.

**Design, Setting, and Participants:**

We retrospectively assessed the use of chromosome 17 p-arm and q-arm alternative control genomic sites (*TP53*, D17S122, SMS, *RARA*, *TOP2A*), as recommended by the 2013/2014 ASCO-CAP guidelines for *HER2* testing, in patients whose data were available through Molecular Taxonomy of Breast Cancer International Consortium (METABRIC) and whose tissues were available through the Breast Cancer International Research Group clinical trials. We used data from an international cohort database of invasive breast cancers (1980 participants) and international clinical trial of adjuvant chemotherapy in invasive, node-positive breast cancer patients.

**Main Outcomes and Measures:**

The primary objectives were to (1) assess frequency of heterozygous deletions in chromosome 17 genomic sites used as FISH internal controls for evaluation of *HER2* status among *HER2*-equivocal cancers; (2) characterize impact of using deleted sites for determination of *HER2*-to-internal-control-gene ratios; (3) assess *HER2* protein expression in each subgroup; and (4) compare clinical outcomes for each subgroup.

**Results:**

Of the 1980 patients in METABRIC,1915 patients were fully evaluated. In addition, 100 *HER2*-equivocal breast cancers by FISH and 100 comparator FISH-negative breast cancers from the BCIRG-005 trial were analyzed. Heterozygous deletions, particularly in specific p-arm sites, were common in both *HER2*-amplified and *HER2*-not-amplified breast cancers. Use of alternative control probes from these regions to assess *HER2* by FISH in *HER2*-equivocal as well as *HER2*-not-amplified breast cancers resulted in high rates of false-positive ratios (*HER2*-to-alternative control ratio ≥2.0) owing to heterozygous deletions of control p-arm genomic sites used in ratio denominators. Misclassification of HER2 status was observed not only in breast cancers with ASCO-CAP equivocal status but also in breast cancers with an average of fewer than 4.0 *HER2* copies per tumor cell when using alternative control probes.

**Conclusions and Relevance:**

The indiscriminate use of alternative control probes to calculate *HER2* FISH ratios in *HER2*-equivocal breast cancers may lead to false-positive interpretations of HER2 status resulting from unrecognized heterozygous deletions in 1 or more of these alternative control genomic sites and incorrect *HER2* ratio determinations.

## Key Points

**Questions:**

How does one assess the status of *HER2* ISH-equivocal breast cancers as either *HER2* positive or *HER2* negative for treatment purposes, and are the use of alternative controls, as recommended by the 2013/2014 American Society of Clinical Oncology and College of American Pathologists guidelines, appropriate?

**Findings:**

In this study, chromosome 17 p-arm genomic sites had a high rate of heterozygous deletions, both in the publicly available Molecular Taxonomy of Breast Cancer International Consortium database and in the Breast Cancer International Research Group-005 clinical trial samples using fluorescence in situ hybridization.

**Meaning:**

The indiscriminate use of alternative controls to assess *HER2* status with *HER2*-to-control gene ratios by ISH may lead to false-positive determinations and should be avoided, as recommended by the 2018 clinical practice update.

## Teaser

This study examines *HER2* ISH-equivocal breast cancers to asses 2013/2014 American Society of Clinical Oncology and College of American Pathologists guidelines in determining *HER2*-positive or *HER2*-negative breast cancers and false-positive results.

## Introduction

Amplification/overexpression of human epidermal growth factor receptor 2 gene (*ERBB2,* formerly *HER2*), is associated with shortened disease-free (DFS) and overall survival (OS) in patients whose breast cancers contain this alteration.^1,2,3^ Because targeted therapies using anti-HER2 humanized monoclonal antibodies,^4,5,6,7^ small molecule inhibitors of HER2 kinase,^8,9,10^ and antibody-drug conjugates^11,12^ effectively treat patients with *HER2*-positive breast cancer, accurate assessment of *HER2* status is critically important for treatment selection. Since HER2 protein overexpression is a direct consequence of *HER2* amplification, a variety of companion diagnostics are used to identify patients for targeted therapy. During the past decade, the American Society of Clinical Oncology (ASCO) and College of American Pathologists (CAP) have specified criteria for clinical assessment of *HER2* amplification status.^13,14,15,16^

The most recent full ASCO-CAP guidelines for HER2 testing by in situ hybridization (ISH) changed the evaluation for *HER2* amplification requiring formalized assessment of both average *HER2* gene number per tumor cell and ratio of average *HER2*-to-internal control chromosome 17 centromere (CEP17) for assessment of *HER2* status by fluorescence in situ hybridization (FISH).^13,14^ This scoring algorithm identifies 5 different breast cancer FISH groupings using *HER2* FISH ratio and average *HER2* copy number per nucleus.^13,14,17,18^ One of these 5 ASCO-CAP FISH groups, designated as ASCO-CAP FISH group 4,^17,18^ is considered “*HER2*-equivocal” (neither amplified nor not-amplified).^13,14^ According to the guidelines, this ambiguous status may be resolved with alternative controls to replace CEP17 for assessment of *HER2* FISH ratios using genes other than CEP17. This approach was widely adopted by both commercial testing laboratories and academic centers. As described,^19,20,21,22^ when any of these alternative control probes leads to a *HER2*-to-control ratio of 2.0 or more, the breast cancer is designated “ISH-positive.”

This approach to *HER2*-equivocal breast cancers contrasts with our experience using alternative controls.^23,24^ Based on early studies of control genes on chromosome 17^2,3,25^ and preliminary findings from the Breast Cancer International Research Group/Translational Research in Oncology (BCIRG/TRIO) central laboratories, we hypothesized that use of chromosome 17 p-arm controls to establish *HER2* gene status of ISH-equivocal breast cancers could lead to false-positive classifications in a significant proportion of cases. In this study, we evaluate these hypotheses: (1) genetic loci used for alternative control probes are heterozygously deleted in a substantial proportion of human breast cancers, especially ISH-equivocal cancers; (2) use of these loci for FISH assessment of *HER2* status leads to *HER2*-to-control ratios greater than or equal to 2.0 and, therefore, false-positive assessments of *HER2* status; and (3) these *HER2* false-positive breast cancers have outcomes that do not differ from behavior established for *HER2*-negative breast cancers.

## Methods

Our study was conducted in 3 parts. The first involved analyses of Molecular Taxonomy of Breast Cancer International Consortium (METABRIC) breast cancer data sets to determine relative frequency of deletions in chromosome regions corresponding to probes used as alternative controls on the chromosome 17 p-arm (*LIS1*, *TP53*, D17S122, *RAI1*, and SMS [Smith-Magenis syndrome] region) and q-arm (*TOP2A* and *RARA*). The second part involved reassessment of *HER2* status in breast cancers from women accrued to BCIRG-005 (ClinicalTrials.gov Identifier NCT00312208) whose cancers had *HER2*-to-CEP17 FISH ratios of less than 2.0 and an average of 4.0 to 5.99 *HER2* genes per tumor cell (ISH-equivocal) or cancers with FISH ratios of less than 2.0 and average *HER2* gene copies less than 4.0 per tumor cell (ISH-negative) using alternative control probes to reassess *HER2* status, according to the 2013/2014 ASCO-CAP guidelines.^19,20,22,26^ Finally, we assessed clinical outcomes in these subgroups.

### Analyses of METABRIC Data Set

Publicly available breast cancer genomic copy number data sets, including 1980 patients from the METABRIC^27^ cohort (European Genome-phenome Archive, https://ega-archive.org/dacs/EGAC00001000484) have been profiled on the Affymetrix single-nucleotide polymorphism (SNP) 6.0 array, which includes more than 906 600 SNPs and more than 946 000 copy number probes. Corresponding clinical data were obtained, including tumor estrogen receptor α/progesterone receptor (ER/PR) status, *HER2* evaluation by IHC (most samples) or FISH. Linear copy number values for *HER2* and alternative probes were compared, as were their copy number states as inferred based on Genomic Identification of Significant Targets in Cancer (GISTIC, version 2.0.23) (https://www.ncbi.nlm.nih.gov/pubmed/21527027/) using default settings. In addition, allele-specific copy number analysis of tumors (ASCAT)^28^ total allele counts (corrected for ploidy and purity) were obtained for the METABRIC cohort from Pereira et al.^29^ To define the alternative probe regions of interest (*LIS1*, *TP53*, D17S122, *RAI1*, SMS, *TOP2A,* and *RARA*), each region was delineated by the position of the gene/genes contained (D17S122 contains *CDRT7*, *PMP22*, *TEKT3*, *CDRT4,* and *TVP23C*; SMS contains *LLGL1*, *FLII*, *TOP3A,* and *SHMT1*; *RARA*-*TOP2A* contains *RARA* and *TOP2A*). Individual cells were compared by selecting a representative gene from each region (*TEKT3* for D17S122, *TOP3A* for SMS, and *TOP2A* for RARA-TOP2A).

### Patients From the BCIRG-005 Trial

**Figure 1.**
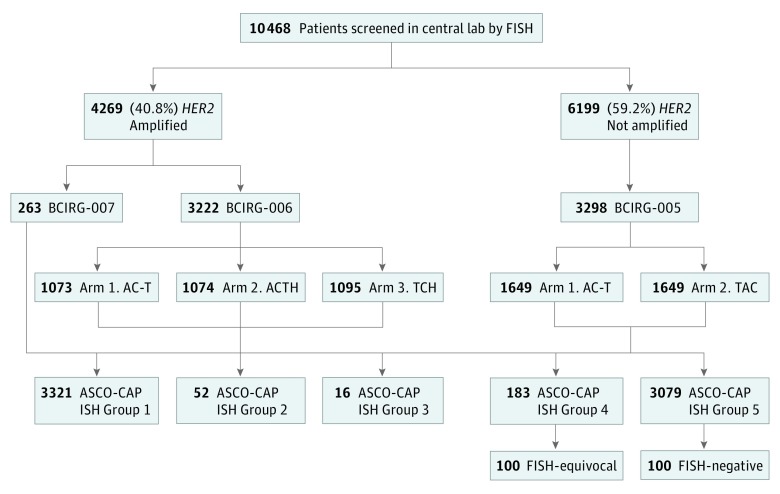
Participant Flow Diagram and Specimen Accountability Breast cancers from patients were evaluated in 1 of 2 central laboratories (laboratory) as either human epidermal growth factor receptor 2 gene *HER2*-not-amplified or *HER2*-amplified for eligibility to 1 of 3 concurrently conducted clinical trials (BCIRG-005, BCIRG-006, and BCIRG-007). One of the trials, BCIRG-005, required patients whose breast cancers were *HER2*-not-amplified and the other 2 trials, BCIRG-006 and BCIRG-007, required patients whose breast cancers were *HER2*-amplified, as determined with fluorescent in situ hybridization (FISH). Although 10 948 patients were screened in the Breast Cancer International Research Group central laboratories for trial accrual, complete *HER2* FISH assay results were available from 10 468 patients for a variety of reasons, including lack of invasive carcinoma in samples submitted, tissue sections that detached from slides during processing, and FISH assay failure owing to lack of probe hybridization. BCIRG-005 randomized patients with *HER2*-not-amplified breast cancers to sequential (arm 1) or concurrent (arm 2) anthracycline, cyclophosphamide, and docetaxel chemotherapy. BCRIG-006 randomized patients with *HER2*-amplified breast cancers to standard anthracycline-containing chemotherapy (arm 1, AC-T) alone, AC-T with trastuzumab (arm 2, ACTH) or a nonanthracycline chemotherapy regimen with trastuzumab (arm 3, TCH). The breast cancers from these trials were subsequently pooled according to the ASCO-CAP guidelines for HER2 testing by FISH as recommended into 5 in situ hybridization (ISH) groups, identified in the lower portion of the figure (ASCO-CAP ISH group 5, ASCO-CAP ISH group 4, ASCO-CAP ISH group 3, ASCO-CAP ISH group 2, and ASCO-CAP ISH group 1) and reanalyzed for correlations with HER2 protein expression and clinical outcomes.^17^ Since ASCO-CAP ISH group 4 is composed exclusively of *HER2*-equivocal breast cancers by FISH, the focus of the current investigation, group 4 served as the source of all *HER2*-equivocal breast cancers (N = 100) characterized in this study by FISH with alternative control probes. As a comparator group, ASCO-CAP ISH group 5, breast cancers were selected for similar analyses by FISH using the same alternative control probes (N = 100). AC-T indicates anthracycline, cyclophosphamide, and docetaxel; ACTH, anthracycline, cyclophosphamide, docetaxel, and trastuzumab; TAC, taxotere, docetaxel, and cyclophosphamide; TCH, docetaxel, carboplatin, and trastuzumab.

Because the second and third parts of our current study were reassessments of breast cancers designated as ISH-equivocal and ISH-negative by 2013/2014 ASCO-CAP FISH guidelines and because such cases were accrued to the BCIRG-005 trial, our focus was on this trial^30,31^ (Figure 1) (Supplement). This randomized trial of concurrent TAC or sequential AC-T adjuvant anthracycline-containing chemotherapy demonstrated the regimens were equally efficacious but differed in levels of toxic effects.^30,31^ This trial was previously approved by human investigations committees of each institution that accrued patients. Written informed consent was obtained from each patient. The central laboratory obtained institutional review board approval for the characterization of HER2 status of tumor samples from each patient.

We included all patients whose breast cancers were both ISH-equivocal by standard FISH (*HER2*/CEP17) (183 participants) (Figure 1) and whose breast cancers were successfully hybridized with all alternative controls used in this study to replace CEP17 (*TP53*, SMS, D17S122, *RARA*, and *TOP2A*) (100 participants) (Figure 1) to recalculate the *HER2* FISH ratio, as recommended.^13,14,19,21,22^ This portion of our study is based on 100 ISH-equivocal and 100 ISH-negative cases from BCIRG-005 successfully reanalyzed with 5 alternative controls by FISH. For comparison with ISH-equivocal, we selected 100 *HER2*-not-amplified breast cancers that, according to the guidelines, are ISH-negative (our ASCO-CAP ISH group 5 breast cancers^17,18^) beginning with those samples that had an average *HER2* gene copy number just less than 4.0 and selected every case in sequential order beginning with 3.99 copies per tumor cell until we had a comparison group of 100 cases that also had successful hybridization with all alternative control probes used in the study (100 participants) (Figure 1).

### Laboratory Methods

Tissue sections^6,17,32^ or tissue microarrays^33^ previously analyzed for *HER2* gene amplification status were rehybridized and reanalyzed using alternative control chromosome 17 probes (*TP53*, D17S122, *RAI1*, SMS, *TOP2A*, and *RARA*) by FISH. The primary invasive breast carcinomas of these patients were previously analyzed for HER2 protein expression using immunohistochemical analysis.^17,18,32^ Among these, 80 ISH-equivocal and 100 ISH-negative cases had HER2 immunohistochemical results available for comparison (Supplement).

#### HER2 FISH Assays 

*HER2* FISH assays were performed using the PathVysion assay (Abbott-Molecular, Inc) (Supplement).^17,18,32,34,35^ We characterized *HER2*-equivocal and *HER2*-negative breast cancers by FISH with alternative controls (*TP53*, D17S122, SMS, *TOP2A*, *RARA*) according to current full (2013/2014) ASCO-CAP guidelines^13,14^ (Figure 1) (eFigure 1 in the Supplement).

#### HER2 Protein Expression by Immunohistochemistry

The HercepTest (Dako) as well as a laboratory-developed HER2 10H8-IHC assay were used to evaluate HER2 protein expression in the BCIRG-005 trial (Supplement).^17,18,32,35^

### Statistical Methods

A 2-tailed Fisher exact test was used to compare the frequencies of deletions or gains among q and p chromosome arms using the *fisher.test* function in the R Statistical Programming language (version, 3.4.4; R Foundation, Inc); 95% confidence intervals are reported. A Mann-Whitney test was used for comparing copy number values among different regions at chromosome 17p using the *wilcox.test* function in R. The Mann-Whitney was selected because copy number data do not follow a gaussian distribution. The Spearman correlation among copy number values at chromosome 17p was calculated with *cor* function in R statistical software. Log-rank tests were used to compare BCIRG-005 DFS and OS between different subgroups.

## Results

To evaluate heterozygous deletions of chromosome 17 p-arm and q-arm genomic sites, we used publicly available SNP array data from the METABRIC data set, and confirmed those findings using the same genetic loci by FISH in BCIRG-005 breast cancer specimens.

### Evaluation of Heterozygous Deletions in Alternative Chromosome 17 Genomic Regions Based on METABRIC

**Figure 2.**
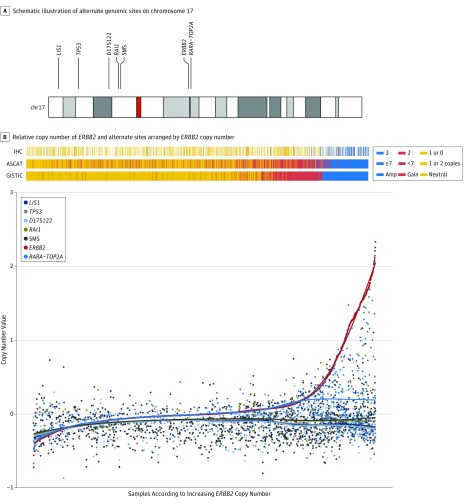
Relative Copy Number of *ERBB2* and Genomic Sites Used as Alternate Controls for Assessment of HER2 Status by FISH (METABRIC Cohort, SNP Chip Data for 1980 Patients) A, Schematic illustration of the positions of alternative control genomic sites (p-arm: *LIS1*, *TP53*, D17S122, *RAI1*, SMS; and q-arm: *RARA*, *TOP2A*) relative to *ERBB2/HER2* on chromosome 17. The location of chromosome 17 centromere is highlighted in red. B, Relative copy number of *ERBB2/HER2* and genomic sites used as alternative controls for assessment of HER2 Status by FISH (METABRIC SNP array data for 1980 patients). Samples were ordered by their *HER2* CN value and plotted alongside the copy number profiles for alternative probes. A linear regression line was fit for each gene (probe). Top bar shows annotations for samples based on IHC, ASCAT, and GISTIC.

We identified genomic regions of chromosome 17 reported as alternative control sites for assessment of *HER2* FISH ratios.^19,20,21^ These sites were assessed in METABRIC for copy number gains and losses relative to *ERBB2/HER2* gains and losses (Table 1) (Figure 2) (eFigure 1 in the Supplement).

**Table 1.** Chromosome 17 Regional Gene Copy Gains and Losses Based on GISTIC Among Alternative Control Genomic Sites Compared With *ERBB2/HER2* Gene Copy Gains and Losses in the METABRIC Cohort Including 1915 Participants^a^

Alternative Control (region)	*ERBB2/HER2* Gene Copy No. Status, No. (%)				Total
	*HER2* Loss	*HER2* Normal	*HER2* Gain	*HER2* Amp	
***LIS1***					
Gain	9 (2.5)	25 (2.7)	61 (18.7)	13 (4.5)	108 (5.6)
Normal	46 (12.7)	622 (66.5)	64 (19.6)	87 (29.9)	819 (42.8)
Loss	308 (84.8)	288 (30.8)	201 (61.7)	191 (65.6)	988 (51.6)
Total	363 (100)	935 (100)	326 (100)	291 (100)	1915 (100)
***TP53***					
Gain	3 (0.8)	15 (1.6)	60 (18.4)	4 (1.4)	82 (4.3)
Normal	41 (11.3)	624 (66.7)	59 (18.1)	81 (27.8)	805 (42.0)
Loss	319 (87.9)	296 (31.7)	207 (63.5)	206 (70.8)	1028 (53.7)
Total	363 (100)	935 (100)	326 (100)	291 (100)	1915 (100)
**D17S122 (*TEKT3*)**					
Gain	10 (2.7)	11 (1.2)	55 (16.9)	8 (2.7)	84 (4.4)
Normal	50 (13.8)	643 (68.8)	67 (20.5)	84 (28.9)	844 (44.1)
Loss	303 (83.5)	281 (30.0)	204 (62.6)	199 (68.4)	987 (51.5)
Total	363 (100)	935 (100)	326 (100)	291 (100)	1915 (100)
***RAI1***					
Gain	7 (1.9)	39 (4.2)	78 (23.9)	35 (12.0)	159 (8.3)
Normal	57 (15.7)	640 (68.4)	64 (19.9)	89 (30.6)	851 (44.4)
Loss	299 (82.4)	256 (27.4)	183 (56.1)	167 (57.4)	905 (47.3)
Total	363 (100)	935 (100)	326 (100)	291 (100)	1915 (100)
**SMS (*TOP3A*)**					
Gain	12 (3.3)	42 (4.5)	79 (24.2)	41 (14.1)	174 (9.1)
Normal	54 (14.9)	647 (69.2)	71 (21.8)	94 (32.3)	866 (45.2)
Loss	297 (81.8)	246 (26.3)	176 (54.0)	156 (53.6)	875 (45.7)
Total:	363 (100)	935 (100)	326 (100)	291 (100)	1915 (100)
**TOP2A/RARA (*TOP2A*)**					
Gain	2 (0.5)	19 (2.0)	284 (87.1)	117 (40.2)	422 (22.0)
Normal	22 (6.1)	899 (96.1)	36 (11.0)	77 (26.5)	1034 (54.0)
Loss	339 (93.4)	17 (1.8)	6 (1.8)	97 (33.3)	459 (24.0)
Total	363 (100)	935 (100)	326 (100)	291 (100)	1915 (100)

Abbreviations: D17S122, the 17p-arm genomic locus which is duplicated in Charcot-Marie-Tooth disease; GISTIC, genomic identification of significant targets in cancer; *HER2*, human epidermal growth factor receptor 2 gene; METABRIC, Molecular Taxonomy of Breast Cancer International Consortium; *RARA*, retinoic acid receptor-alpha gene; SMS, Smith-Magenis Syndrome locus; *TOP2A*, topoisomerase-II-alpha gene.

^a^ Genomic Identification of Significant Targets in Cancer, a tool to identify genes targeted by somatic copy-number alterations; note that a subset of cases was not evaluable by GISTIC, thus 1915 of 1980 cases are reported here.

Homozygous deletion of any alternative control site was distinctly unusual; however, heterozygous deletions were relatively common, especially on the p-arm of chromosome 17. Alternative control sites on the p-arm showed a higher rate of heterozygous deletion than q-arm alternative control sites. A scatterplot of *ERBB2/HER2* linear copy numbers compared with alternate control sites (*LIS1*, *TP53*, D17S122, *RAI1*, SMS, *TOP2A*, and *RARA*) demonstrated relatively high rates of gene copy loss using the alternative control genomic sites located on the p-arm (Table 1) and a significantly lower rate of loss among q-arm markers (*P* < .001; OR, 0.29; 95% CI, 0.25-0.34; Fisher exact test for the frequency of *TOP2A* [q-arm] was lower than the frequency of *TETK3* [p-arm] in the METABRIC cohort) (Table 1) (Figure 2B) (eFigure 1 in the Supplement). Deletions in *LIS1*, *TP53*, D17S122, *RAI1*, and SMS were observed in 288 (31%), 296 (32%), 281 (30%), 256 (27%), and 246 (26%) of 935 cases, respectively, with concurrent *HER2* normal copy numbers (Table 1). In contrast, only 17 (1.8%) of 935 METABRIC samples showed loss of *RARA* and *TOP2A* (q-arm) with a normal *HER2* copy number state (Table 1).

In METABRIC, 32% of samples demonstrated either a copy number gain or amplification of *ERBB2/HER2* with 15% of samples showing high-level gains, considered amplifications. We considered those breast cancers with *HER2* copy number “gain” but not “high gain” (amplification) to be most likely representative of ISH-equivocal cancers with increased *HER2* (4-6 copies per tumor cell), but not *HER2* amplification. Among those with *HER2* copy number gain but not amplification, in the p-arm *LIS1*, *TP53*, D17S122, *RAI1*, and SMS regional losses occurred in 201 (62%), 207 (64%), 204 (63%), 183 (56%), and 176 (54%) of 326 cases, respectively (Table 1) (Figure 2B) (eFigure 1 in the Supplement). Regional loss on the q-arm (*TOP2A* and *RARA* genes) was significantly less frequent (1.84%) (*P* < .001; OR, 0.01 [95% CI, 0.003-0.025]; Fisher exact test for *TETK3* in p-arm and *TOP2A* in q-arm).

The rate of p-arm alternative control region deletions was even higher among breast cancers with high-level gain or amplification than in the *HER2* copy gain cancers (Table 1) (Figure 2B) (eFigure 1 in the Supplement). Therefore, more than half of the samples with *HER2* gain or amplification showed a loss of p-arm regions.

Comparison of HER2 IHC values revealed that IHC 3^+^ cases in METABRIC exhibited deletions of the alternative control probe loci (eFigure 1B in the Supplement). A similar deletion pattern in the control probes were observed when using the total count of alleles as estimated by ASCAT (correcting by ploidy and purity) (eFigure 1C in the Supplement). Differences were noted between alternative probes located in 17p-arm such that genes located at 17p13-12 (*LIS1*, *TP53,* and D17S122) were more frequently deleted than genes at 17p11 (DSS and *RAI1*) (*P* < .001; θ = −0.0261; 95% CI, −0.0351 to 0.0171; Mann-Whitney test for *TP53* vs DSS in METABRIC). This difference was magnified in amplified samples (*P* < .001; θ = −0.0533; 95% CI, −0.077 to −0.0299; Mann-Whitney test in METABRIC amplified samples). The negative correlation between *HER2* copy-number state (loss, neutral, gain) and alternative probe values was also higher in the 17p13-12 region (*LIS1*, *TP53,* and D17S122) compared with 17p11 (*RAI1* and SMS) (Spearman correlation ρ = −0.142 [*P* < .001], −0.158 [*P* < .001], −0.172 [*P* < .001], vs −0.078 [*P* < .001], −0.05 [*P* = .03] in METABRIC).

### Chromosome 17 Alternative Control Regions Demonstrate Heterozygous Deletion by FISH

Among 100 ASCO-CAP FISH group 4 (*HER2*-equivocal) breast cancers, we identified heterozygous deletions (Table 2) (eTable 1 and eFigure 2 in the Supplement) in 65 for the SMS locus, 46 for D17S122, 43 for *TP53*, 8 for *TOP2A*, none for *RARA*, and none for *HER2* (eTable 1 in the Supplement). The p-arm alternative control loci had a significantly higher rate of deletions relative to q-arm loci (eTable 1 in the Supplement)(*P* < .001) with SMS the most frequently lost, followed by D17S122 and *TP53*. The frequency of q-arm heterozygous deletions among *HER2*-equivocal cancers was low, with only *TOP2A* reaching 8%. Using the observed average *HER2* copy number (varied from 4 to 5.99) per tumor cell divided on a case-by-case basis by the corresponding observed average alternative control copy numbers demonstrated that ratios greater than or equal to 2.0 were observed for 61 cases using SMS, 65 using *TP53*, and 30 using D17S122 as the denominators in the *HER2*-to-control ratio calculations. Use of *RARA* or *TOP2A* resulted in fewer cases with *HER2*-to-control ratios greater than 2.0 (7% and 25%) (eTable 1 in the Supplement).

**Table 2.** Criteria for Evaluation of Heterozygous Deletions at Alternative Control Genomic Sites on Chromosome 17 by FISH

Chromosome 17 Arm	Gene / Locus	Ratio	Interpretation	Ratio	Interpretation
p-arm	SMS	<0.75^a^	SMS with heterozygous deletion relative to *RARA*	>1.25^b^	*RARA* with heterozygous deletion relative to SMS
q-arm	*RARA*				
p-arm	*TP53*	<0.75^c^	*TP53* with heterozygous deletion relative to *TOP2A*	>1.25^d^	*TOP2A* with heterozygous deletion relative to *TP53*
q-arm	*TOP2A*				
p-arm	D17S122	<0.75^e^	D17S122 with heterozygous deletion relative to *HER2*	>1.25^f^	*HER2* with heterozygous deletion relative to D17S122
q-arm	*HER2*				

Abbreviations: D17S122, the 17p-arm genomic locus which is duplicated in Charcot-Marie-Tooth disease; FISH, fluorescence in situ hybridization; *HER2*, human epidermal growth factor receptor 2 gene.; *RARA*, retinoic acid receptor-alpha gene; SMS, Smith-Magenis syndrome locus; *TOP2A*, topoisomerase-II-alpha gene; *TP53*, tumor protein 53 tumor suppressor gene.

^a^ Subjective assessment of signals also requires the observation that SMS signals are loosely paired with *RARA* signals plus additional individual, scattered *RARA* signals. These paired and individual signals are scattered randomly within tumor cell nuclei, not clustered or aggregated as observed with amplified genes, illustrated in eFigure 2 in the Supplement.

^b^ Subjective assessment of signals also requires the observation that most *RARA* signals are loosely paired with SMS signals plus additional individual, scattered SMS signals. These paired and individual signals are scattered randomly within tumor cell nuclei, not clustered or aggregated as observed with amplified genes.

^c^ Subjective assessment of signals also requires observation of *TP53* signals loosely paired with *TOP2A* signals plus excess additional *TOP2A* signals. These paired and individual signals are scattered randomly within tumor cell nuclei, not clustered or aggregated as observed with amplified genes, illustrated in eFigure 2 in the Supplement.

^d^ Subjective assessment of signals also requires the observation that most *TOP2A* signals are loosely paired with *TP53* signals. These paired and individual signals are scattered randomly within tumor cell nuclei, not clustered or aggregated as observed with amplified genes.

^e^ Subjective assessment of signals also requires the observation that D17S122 signals are loosely paired with *HER2* signals plus excess unpaired *HER2* signals. These paired and individual signals are scattered randomly within tumor cell nuclei, not clustered or aggregated as observed with amplified genes, illustrated in eFigure 2 in the Supplement.

^f^ Subjective assessment of signals also requires the observation that most *HER2* signals are loosely paired with D17S122 signals. These paired and individual signals are scattered randomly within tumor cell nuclei, not clustered or aggregated as observed with amplified genes.

Among 100 ASCO-CAP FISH group 5 (*HER2*-not-amplified or ISH-negative) breast cancers, we identified heterozygous deletions in 35 cases for D17S122, 30 for SMS, 3 for *TOP2A*, 1 for *RARA*, and none for *TP53* (eTable 1 in the Supplement). The p-arm SMS and D17S122 alternative control loci had significantly higher deletion rates than other loci (eTable 1 in the Supplement) (*P* < .001). The frequency of p-arm heterozygous deletions among *HER2*-negative breast cancers was lower than rates observed among *HER2*-equivocal breast cancers. Nevertheless, use of these alternative controls in ASCO-CAP FISH group 5 breast cancers would lead to significant up-grading of HER2 status to ISH-positive (eTable 1 in the Supplement). Using the observed average *HER2* copy number, which varied from 3.99 to 3.25 per tumor cell divided on a case-by-case basis by the correspondingly observed average alternative control copy number, we demonstrated ratios greater than 2.0 for 37 cases using SMS, 12 with *RARA*, 11 with D17S122, 2 with *TP53*, and 1 with *TOP2A* as the denominators in the *HER2*-to-control ratio calculations. Similar to *HER2*-equivocal breast cancers, the use of heterozygously deleted alternative control loci was associated with *HER2*-to-control ratios equal to or greater than 2.0, consequently, false-positive ratios through status upgrading.

### HER2 Protein Expression and Alternative Control Subgroups

The ASCO-CAP FISH group 4 (*HER2*-equivocal) and ASCO-CAP FISH group 5 (*HER2*-negative) breast cancers included in this study were associated with low levels of HER2 protein expression by immunohistochemical analysis (eTable 2 in the Supplement). Separation of group 4 and group 5 according to FISH ratios using the various alternative controls did not alter this association for any subgroup by ratio greater than or equal to 2.0, determined with any alternative control probe (eTable 2 in the Supplement), consistent with the interpretation that these alternative control ratios of 2.0 or more do not identify a subgroup of cancers which should be considered for upgrading of *HER2* status.

### Clinical Outcomes by Alternative Control Subgroups

**Figure 3.**
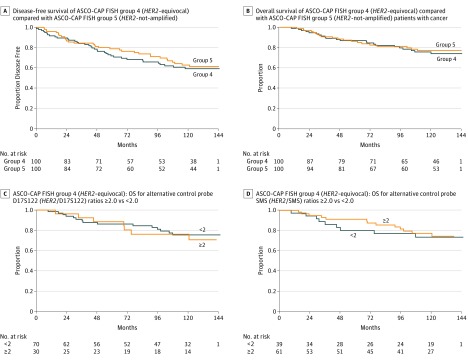
Comparison of Clinical Outcomes for ASCO-CAP Group 4 (*HER2*-Equivocal) and ASCO-CAP Group 5 (*HER2*-Negative) Patients With Breast Cancer A, Disease-free survival of ASCO-CAP FISH group 4 (*HER2*-equivocal) compared with ASCO-CAP FISH group 5 (*HER2*-not-amplified). There was no significant difference in disease-free survival for the 100 patients with ASCO-CAP FISH group 4 (*HER2*-equivocal) breast cancers compared with the 100 with ASCO-CAP FISH group 5 (*HER2*-not-amplified) breast cancer. B, Overall survival of ASCO-CAP FISH group 4 (*HER2*-equivocal) compared with ASCO-CAP FISH group 5 (*HER2*-not-amplified) patients with breast cancer. There was no significant difference in overall survival for the 100 patients with ASCO-CAP FISH group 4 (*HER2*-equivocal) breast cancers compared with the patients with 100 ASCO-CAP FISH group 5 (*HER2*-not-amplified) breast cancer. C, ASCO-CAP FISH group 4 (*HER2*-equivocal): OS for Alternative Control Probe D17S122 (*HER2*/D17S122) ratios ≥2.0 vs <2.0. Among women with *HER2*-equivocal breast cancers with an D17S122 alternative control probe ratio ≥2.0 does not identify a subgroup with a worse overall survival. D, ASCO-CAP FISH group 4 (*HER2*-equivocal): OS for alternative control probe SMS (*HER2*/SMS) ratios ≥2.0 vs <2.0. Among women with *HER2*-equivocal breast cancers, those who had a SMS alternative control probe ratio ≥2.0 appear to have a slightly better overall survival than those whose breast cancers had a SMS alternative control probe ratio <2.0; however, this difference was not significant.

Within the ISH-equivocal breast cancer patients whose cancers were upgraded to ISH-positive using alternative control probes, there was no subgroup of patients who had a significantly worse disease-free or overall survival compared with either the overall ISH-equivocal or ISH-negative patients or those with a *HER2*-to-alternative control probe ratio of less than 2.0 (Figure 3) (eTable 3 and eFigure 3 in the Supplement). Similar observations were made among the patients whose cancers were ISH-negative breast cancer and were upgraded to ISH-positive by the use of alternative control probes.

## Discussion

ISH-equivocal, as defined by the 2013/2014 ASCO-CAP guidelines^13,14^ and the recent 2018 update,^36^ represent approximately 4% to 12% of all breast cancers, or 7000 to 21 000 patients annually.^17,18,20,37,38,39,40,41^ These cases present patients with breast cancer, oncologists, and pathologists some of their most frequent clinical challenges, because questions remain regarding how *HER2* status should be resolved and confusion about HER2-targeted therapies for these patients. Several studies reported the use of alternative controls among patients whose cancers had ISH-equivocal status.^19,20,21,22,26^ In these studies, if any *HER2*-to-alternative probe ratio was 2.0 or more, the breast cancer was upgraded to ISH-positive.^19,20,21,26^

The potential for internal comparator controls to impact assessment of *HER2* status was not addressed by previous studies but has concerned us since we began characterizing this alteration.^2,3,25^ In 2 early investigations of *HER2* amplification using the same 345 breast cancers, we used myeloperoxidase gene (*MPO*) as the internal control (*HER2*-to-*MPO* ratio)^3^ whereas another group used *TP53* (*HER-*to*-TP53* ratio)^42^ for assessment of *HER2* status. The studies came to different conclusions about both amplification frequency in the cohort (27% vs 33%) and associations with clinical outcomes (significantly associated with DFS and OS^3^ vs neither^42^) using the same shared *HER2* gene data by Southern hybridization.^3,42^ The choice of control gene to calculate ratios was the only difference.

The 2013/2014 ASCO-CAP guidelines did not provide data to support use of chromosome 17 alternative probes and the recent 2018 Focused Update did not provide data justifying discontinuation of this approach to resolve the status of ISH-equivocal cancers. Here we used METABRIC data as well as cancers from the BCIRG-005 trial to demonstrate heterozygous deletions among various p-arm and q-arm genomic sites previously used as alternative controls. Our findings indicate heterozygous deletions, particularly at p-arm genomic sites, are relatively common and are not restricted to breast cancers with ISH-equivocal status. In addition, among cancers upgraded by *HER2*-to-alternative-control ratios of 2.0 or greater there was no significant association with either HER2 protein overexpression or worse clinical outcomes. Our conclusion, that ISH-equivocal breast cancers are *HER2*-not-amplified is also supported by our previous data,^17,18^ which demonstrate that ASCO-CAP FISH group 4 (*HER2*-equivocal) breast cancers lack HER2 protein overexpression^17,18^ and have patient outcomes^17^ that are not significantly different from ASCO-CAP FISH group 5 (HER2-negative or *HER2*-not-amplified) breast cancer patients. These findings are consistent with the perspective that *HER2*-equivocal cancers upgraded to *HER2*-positive are the result of heterozygously deleted alternative control probes. These findings do not mean use of alternative controls for assessment of amplification has no value, only that selection of internal controls should be done carefully with the possibility of heterozygous deletions clearly considered. In our laboratory we have established criteria for the use of such alternative control probes (Table 2) (eFigure 2 in the Supplement).

Because p-arm genomic sites frequently have heterozygous loss, using a *HER2*-to-p-arm alternative control ratio in ISH-equivocal breast cancers can lead to overestimation of the *HER2*-amplification status. There is a lack of evidence to support the view that *HER2* upgraded, ISH-equivocal breast cancers have a clinical disease similar to *HER2*-amplified breast cancers. We and others^20^ show that women with ISH-equivocal cancers upgraded to HER2-positive by p-arm alternative controls do not have significantly worse outcomes than those who are not upgraded. ISH-equivocal cancer patients show outcomes similar to those with ISH-negative disease with no difference in DFS or OS that warrants upgrading to ISH-positive status. We predict lack of responsiveness to *HER2*-targeted therapies among these patients whose cancers lack *HER2*-amplification, consistent with previous trials of lapatinib^9^ and, more recently, the NSABP-B47 trial of adjuvant trastuzumab in 3270 patients with breast cancers having weakly positive *HER2* expression by IHC (1^+^) or moderately positive *HER2* expression by IHC (2^+^) but *HER2*-not-amplified (*HER2*-negative) by FISH.^43^ Therefore, use of HER2-directed therapies in a population falsely classified as *HER2*-positive is expected to produce inferior clinical and pharmacoeconomic outcomes.

### Limitations

The study has some noteworthy limitations. Although a large number of breast cancer cases were available from the METABRIC database for analyses, *HER2* gene copy number estimates were based on SNP array data, not *HER2* gene copy numbers determined by FISH. In contrast, *HER2* gene copy number data were available from the BCIRG-005 trial; however, the number of patients in this trial with long-term clinical follow-up who had breast cancers with *HER2* FISH-equivocal (ASCO-CAP FISH group 4) status was limited.

## Conclusions

Assessment of *HER2* gene status in *HER2*-equivocal breast cancers is important and clinically relevant. Although approximately half of such *HER2*-equivocal cases have been upgraded to *HER2*-positive by FISH using alternative control probes, deletions in these alternative control genomic sites on chromosome 17 can lead to false-positive *HER2*-to-control ratios (greater than or equal to 2.0). We show the genomic sites used to assess these *HER2*-to-internal control ratios, especially those on the p-arm of chromosome 17, have frequent heterozygous deletions. These deletions, when used for assessment of *HER2*-equivocal (ASCO-CAP FISH group 4) breast cancers lead to frequent false-positive *HER2*-to-internal control ratios greater than 2.0. *HER2*-equivocal breast cancers with these false-positive ratios do not have HER2 protein overexpression and these patients do not have clinical outcomes that differ from either other patients with *HER2*-equivocal breast cancers not upgraded to positive or from *HER2*-negative disease. Our findings indicate that *HER2* FISH-equivocal breast cancers are *HER2*-not-amplified.

## Notes

Supplement. **eIntroduction** **eMaterials and Methods** **eDiscussion** **eTable 1.** Assessment of Heterozygous Deletions by FISH using Pairwise Comparisons of Alternative Control Genes and Frequency of resulting HER2 FISH ratios greater than 2.0 using these same Alternative Control Genes: ASCO-CAP Group 4 (HER2-Equivocal) and ASCO-CAP Group 5 (HER2-not-amplified) Breast Cancers **eTable 2.** Correlation of HER2 Protein Status by IHC among BCIRG-005 Trial Breast Cancers determined to be “HER2-Positive” using Various Alternative Control Probes (SMS, D17S122 and TP53) among ASCO-CAP Group 4 (HER2-Equivocal) and ASCO-CAP Group 5 (HER2-not-amplified) Breast Cancers **eTable 3.** Outcomes for BCIRG-005 Trial Patients whose Breast Cancers were “HER2-Positive” using Various Alternative Control Probes (SMS, D17S122 and TP53) among ASCO-CAP Group 4 (HER2-Equivocal) and ASCO-CAP Group 5 (HER2-not-amplified) Breast Cancers **eFigure 1.** Relative copy number of HER2 / ERBB2 and Genomic Sites (LIS1, TP53, D17S122, RAI1 SMS, RARA-TOP2A) used as Alternate Controls for Assessment of HER2 Status by FISH (METABRIC COHORT. SNP chip data; N = 1980) **eFigure 2.** Assessment of Heterozygous Deletion Among Chromosome 17 genomic sites by comparison of p-arm (TP53, SMS, D17S122) with q-arm (RARA, TOP2A, HER2) probes. Breast cancers with similar numbers of p-arm and q-arm markers are interpreted as showing a lack of deletion at those specific genomic sites, while those with an imbalance between p-arm and paired q-arm marker are considered to have heterozygous deletion **eFigure 3.** Comparison of Clinical Outcomes for ASCO-CAP Group 4 (HER2-Equivocal) and ASCO-CAP Group 5 (HER2-negative) Breast Cancer Patients. Kaplan-Meier plots **eReferences**
