# Supplementary material for: Assessment of ERBB2/HER2 Status in HER2-Equivocal Breast Cancers by FISH and 2013/2014 ASCO-CAP Guidelines
Source: JAMA Oncol. 2018 Dec 6;5(3):366–75. doi: 10.1001/jamaoncol.2018.6012 (PMC6439848; doi:10.1001/jamaoncol.2018.6012)
Supplement: Supplement. — eIntroduction eMaterials and Methods eDiscussion eTable 1. Assessment of Heterozygous Deletions by FISH using Pairwise Comparisons of Alternative Control Genes and Frequency of resulting HER2 FISH ratios greater than 2.0 using these same Alternative Control Genes: ASCO-CAP Group 4 (HER2-Equivocal) and ASCO-CAP Group 5 (HER2-not-amplified) Breast Cancers eTable 2. Correlation of HER2 Protein Status by IHC among BCIRG-005 Trial Breast Cancers determined to be “HER2-Positive” using Various Alternative Control Probes (SMS, D17S122 and TP53) among ASCO-CAP Group 4 (HER2-Equivocal) and ASCO-CAP Group 5 (HER2-not-amplified) Breast Cancers eTable 3. Outcomes for BCIRG-005 Trial Patients whose Breast Cancers were “HER2-Positive” using Various Alternative Control Probes (SMS, D17S122 and TP53) among ASCO-CAP Group 4 (HER2-Equivocal) and ASCO-CAP Group 5 (HER2-not-amplified) Breast Cancers eFigure 1. Relative copy number of HER2 / ERBB2 and Genomic Sites (LIS1, TP53, D17S122, RAI1 SMS, RARA-TOP2A) used as Alternate Controls for Assessment of HER2 Status by FISH (METABRIC COHORT. SNP chip data; N = 1980) eFigure 2. Assessment of Heterozygous Deletion Among Chromosome 17 genomic sites by comparison of p-arm (TP53, SMS, D17S122) with q-arm (RARA, TOP2A, HER2) probes. Breast cancers with similar numbers of p-arm and q-arm markers are interpreted as showing a lack of deletion at those specific genomic sites, while those with an imbalance between p-arm and paired q-arm marker are considered to have heterozygous deletion eFigure 3. Comparison of Clinical Outcomes for ASCO-CAP Group 4 (HER2-Equivocal) and ASCO-CAP Group 5 (HER2-negative) Breast Cancer Patients. Kaplan-Meier plots eReferences [file jamaoncol-5-366-s001.pdf]

Supplementary Online Content

Press MF, Seoane JA, Curtis C, et al. Assessment of *ERBB2/HER2* Status in *HER2*-Equivical Breast Cancers by FISH and 2013/2014 ASCO-CAP Guidelines [published online December 6, 2018]. *JAMA Oncol*. doi:10.1001/jamaoncol.2018.6012

eIntroduction

eMaterials and Methods

eDiscussion

eTable 1. Assessment of Heterozygous Deletions by FISH using Pairwise Comparisons of Alternative Control Genes and Frequency of resulting HER2 FISH ratios greater than 2.0 using these same Alternative Control Genes: ASCO-CAP Group 4 (HER2-Equivocal) and ASCO-CAP Group 5 (HER2-not-amplified) Breast Cancers

eTable 2. Correlation of HER2 Protein Status by IHC among BCIRG-005 Trial Breast Cancers determined to be "HER2-Positive" using Various Alternative Control Probes (SMS, D17S122 and TP53) among ASCO-CAP Group 4 (HER2-Equivocal) and ASCO-CAP Group 5 (HER2-not-amplified) Breast Cancers

eTable 3. Outcomes for BCIRG-005 Trial Patients whose Breast Cancers were "HER2-Positive" using Various Alternative Control Probes (SMS, D17S122 and TP53) among ASCO-CAP Group 4 (HER2-Equivocal) and ASCO-CAP Group 5 (HER2-not-amplified) Breast Cancers

eFigure 1. Relative copy number of HER2 / ERBB2 and Genomic Sites (LIS1, TP53, D17S122, RAI1 SMS, RARA-TOP2A) used as Alternate Controls for Assessment of HER2 Status by FISH (METABRIC COHORT. SNP chip data; N = 1980)

eFigure 2. Assessment of Heterozygous Deletion Among Chromosome 17 genomic sites by comparison of p-arm (TP53, SMS, D17S122) with q-arm (RARA, TOP2A, HER2) probes. Breast cancers with similar numbers of p-arm and q-arm markers are interpreted as showing a lack of deletion at those specific genomic sites, while those with an imbalance between p-arm and paired q-arm marker are considered to have heterozygous deletion

eFigure 3. Comparison of Clinical Outcomes for ASCO-CAP Group 4 (HER2-Equivocal) and ASCO-CAP Group 5 (HER2-negative) Breast Cancer Patients. Kaplan-Meier plots

eReferences

© 2018 Press MF, et al. *JAMA Oncology.*

This supplementary material has been provided by the authors to give readers additional information about their work.

© 2018 Press MF, et al. *JAMA Oncology.*

eIntroduction

Our earlier studies of *HER2* gene amplification as determined by Southern blot analysis[1-3], indicated that selection of the comparator control gene probes requires considerable caution because, at least some of these control genes may occur at loci frequently deleted in breast cancer, especially *TP53*[4]. Therefore, use of these deleted sites could lead to false-positive interpretations based on a (*HER2* / internal control comparator gene) ratio $\geq$2·0 due to reduction of the denominator in the assessment of *HER2*-to-control gene ratios as opposed to an increase in *HER2* gene copy number resulting from bona fide gene amplification. We further predicted that use of alternative control regions for *HER2* testing can result in false-positive ISH status due to heterozygous deletions for at least some breast cancers.

eMaterials and Methods.

Patients and Clinical Trials. Between August, 2000 and March, 2004 primary invasive breast carcinomas from 10,468 patients were evaluated for HER2 status by fluorescence in situ hybridization (FISH) in one of two central laboratories to determine eligibility for enrollment in Breast Cancer International Research Group (BCIRG) clinical trials, as described[5-10]. Because the second part of our current study is a re-assessment of breast cancers designated as "*HER2*-equivocal" by 2013/2014 ASCO-CAP FISH guidelines and because such cases were systematically excluded from BCIRG-006 and -007 trials, our focus in this study is with the BCIRG-005 trial[6, 7]. This randomized trial of concurrent docetaxel, doxorubicin, and cyclophosphamide (TAC) or sequential (AC-T) adjuvant anthracycline-containing chemotherapy in patients with *HER2*-not-amplified, stage II and III breast cancer demonstrated sequential and combination chemotherapy regimens incorporating three drugs were equally efficacious but differed in toxicity profile[6, 7]. This clinical trial was approved by the human investigations committee of each institution that accrued patients to the BCIRG-005 trial. Written informed consent was obtained from each study participant at the institution accruing the patient. The central laboratory obtained institutional review board approval (IRB number: HS-008070) for the characterization of HER2 status of tumor samples from each patient in this study.

This portion of our study is based on 100 "FISH-equivocal" and 100 "FISH-negative" cases from the BCIRG-005 trial re-analyzed with use of alternative control probes by FISH. Among these, 80 "*HER2*-equivocal" and 100 "*HER2*-not-amplified" cases had HER2 immunohistochemistry available for comparison (eTable 2).

Laboratory Methods. *Fluorescence In Situ Hybridization (FISH).* Patients whose breast cancers were *HER2*-amplified, that is, the tumor cells had a *HER2*-to-chromosome 17 centromere (CEP17) FISH ratio $\geq$2.0 without regard for the average *HER2* gene copy number as originally approved by the U.S. Food and Drug Administration (FDA)[11, 12] met an eligibility criterion for BCIRG-006 and BCIRG-007, but were not eligible for BCIRG-005. Whereas those whose breast cancers were composed of tumor cells with a *HER2*-to-chromosome 17 centromere (CEP17) FISH ratio <2.0 without regard for the average *HER2* gene copy number were *HER2*-not-amplified by FDA-approved criteria and met an eligibility criterion for the BCIRG-005 trial. Only tissue samples from these latter patients were used for the current study of "*HER2*-equivocal" breast cancers since these had *HER2*-to-CEP17 ratios <2.0.

As we have reported[8, 13], the BCIRG-005 trial accrued 183 women whose breast cancers had a *HER2*-to-CEP17 FISH ratio <2.0 with an average *HER2* gene copy number $\geq$4.0, but <6.0 per tumor cell nucleus. Of these, 100 were successfully re-evaluated with five different alternative control probes as part of this study. As a comparison group, we also re-evaluated 117 patients whose breast cancers had a *HER2*-to-CEP17 FISH ratio <2.0 with an average *HER2* gene copy number between 3.2 and 3.99 per tumor cell nucleus and were accrued to the BCIRG-005 trial.

This second portion of our report is based on the 200 cases that were successfully analyzed with all alternative control probes by FISH, 100 ASCO-CAP FISH group 4 (*HER2*-equivocal) and 100 ASCO-CAP FISH group 5 (*HER2*-not-amplified) breast cancers. Among these, 80 "*HER2*-equivocal" and 100 "*HER2*-not-amplified" also had HER2 immunohistochemistry available for comparisons.

HER2 FISH Assays. *HER2* FISH assays were performed using the PathVysion assay (Abbott-Molecular, Inc.), as described[5, 8, 11, 13, 14]. We characterized "*HER2*-equivocal" and *HER2*-negative breast cancers by FISH with alternative controls according to current full (2013/2014) ASCO-CAP guidelines[15, 16]. A number of genes located on either the p-arm or q-arm of chromosome 17 (*TP53*, D17S122, SMS, *TOP2A*, *RARA*) were used as alternative controls in place of CEP17 to calculate the *HER2*-to-control ratio to "resolve" HER2 status of "ISH-equivocal" breast cancers.

© 2018 Press MF, et al. *JAMA Oncology.*

We used FISH probes for these chromosome 17 genes to assess HER2 status in ASCO-CAP FISH group 4 "ISH-equivocal" breast cancers and in a similar number of ASCO-CAP FISH group 5 (ISH-negative) breast cancers from BCIRG-005.

Interpretation of HER2 Fluorescence In Situ Hybridization (FISH) Assay Results according to the 2013/2014 ASCO-CAP Guidelines. According to the ASCO-CAP guidelines[15, 16], in situ hybridization (ISH) assay results are separated into five different groups, based on a combination of average *HER2* gene copy number per tumor cell and *HER2*-to-CEP17 ratios. Three of these groups identify breast cancers that are ISH positive, one ISH equivocal, and one ISH negative. Breast cancers with *HER2*-to-CEP17 ratios ≥2.0 are divided in two groups, one with an average *HER2* gene copy number of ≥4.0/tumor cell (our ASCO-CAP FISH group 1) and one with an average *HER2* gene copy number of <4.0/tumor cell (our ASCO-CAP FISH group 2). Breast cancers with *HER2*-to-CEP17 ratios of <2.0 are divided into three additional groups: one with average *HER2* gene copy number of ≥6.0/tumor cell (our ASCO-CAP FISH group 3), which, according to the 2013/2014 ASCO-CAP guidelines for HER2 testing, is also classified as "ISH positive"; another with average *HER2* gene copy number of ≥4.0 but <6.0/tumor cell (our ASCO-CAP FISH group 4), which has been classified as "ISH-equivocal"; and one with breast cancers that contain an average *HER2* gene copy number of <4.0/tumor cell (our ASCO-CAP FISH group 5), which is classified as ISH-negative. According to the 2013/2014 ASCO-CAP guidelines breast cancers in groups 1, 2, and 3 are interpreted as ISH-positive, group 4 as ISH-equivocal, and group 5 as ISH-negative. Treatment with HER2-targeted agents is a dichotomous decision. Patients who have ISH-positive breast cancers are eligible for HER2-targeted therapy and those who have ISH-negative breast cancers are not. Therefore, four of the five FISH groups are associated with clinical treatment options, while ASCO-CAP FISH group 4 ("ISH-equivocal") has no clear course of treatment. These patients have cancers that require further resolution into either the "positive" or "negative" categories. Among other remedies, the 2013/2014 ASCO-CAP guidelines have recommended the use of alternative control probes to replace the number of chromosome 17 copies for calculation of a *HER2*-to-control probe ratio. As described by others[17-20], if the ratio is ≥2.0 using any chromosome 17 alternative control probe, the *HER2* status is assessed as "ISH-positive". This latter evaluation presumes that the alternative controls are a better representation of the average chromosome 17 copy number than CEP17, a hypothesis that we are addressing in this investigation.

Interpretation of HER2 Fluorescence In Situ Hybridization (FISH) Assay Results using Alternative Control Probes According to Internal Laboratory Specifications. Supportive evidence for heterozygous deletion may be obtained by examining both the distribution of FISH probe signals and the relative number of p-arm gene signals compared with q-arm gene signals assessed as a ratio (average *TP53* copy number compared to average *TOP2A* copy number {ratio}, average SMS copy number compared to average *RARA* copy number {ratio}, and D17S122 compared to *HER2* {ratio}) using a strategy previously established for assessments of 1p (1p36 compared to 1q25) and 19q (19q13 compared to 19p13) deletions in oligodendrogliomas[21, 22]. Although various combinations of p-arm and q-arm genomic site markers have been used, these are the combinations used in this study.

Based on previous observations that the vast majority of breast cancers have either a tetraploid or an aneuploid DNA content in the near tetraploid range[23], we considered a ratio of <0.75 or >1.25 to presumptively indicate an imbalance in either the p-arm or q-arm genomic locus consistent with heterozygous deletion, as described below. (There were no breast cancers which showed a complete loss of any of these genomic markers by FISH.) The p-arm signals in cases with heterozygous deletions are not only less numerous than the q-arm probe or the *HER2* signals, but are also characteristically distributed in a loose pairwise fashion with half to three-quarters of the q-arm signals. The remainder of the q-arm signals are randomly distributed throughout the nucleus without a p-arm partner.

Selection of an appropriate internal control for comparison with *HER2* copy number to distinguish gene amplification from copy number aberrations due to chromosome aneusomy or other genomic alterations is important in our laboratory. We have used several different criteria for assessment of gene amplification by FISH. Among breast cancers that lack *HER2* amplification, *HER2* signals are generally scattered randomly throughout the tumor cell nucleus (Supplemental eFigure 2), not grouped together or "clustered" as expected for an amplicon in a homogeneously stained region of a chromosome (see for example Figure 1B and Supplemental Figure S1A, S1B, S1C in *Journal of Clinical Oncology* 34 {29}: 3518-3528, 2016; or Figure 2A, or Figure 3A, 3B, 3C in *Archives of Pathology and Laboratory Medicine* 140 {11}: 1250-1258, 2016). Such scattered *HER2* gene copy number increases are, in our experience with breast cancer cell lines[24], not associated with increased HER2 protein expression or overexpression.

Since internal comparison genes may be deleted in some cancers, we have devised a strategy to identify these cases by FISH. We routinely process each p-arm locus probe with a q-arm locus probe for pairwise comparison of both the copy number and distribution of signals. Characteristically, in breast cancers lacking p-arm deletions, p-arm

© 2018 Press MF, et al. *JAMA Oncology.*

and q-arm signals are of similar number and often distributed in a loosely arranged pairwise fashion with these "pairs" randomly distributed throughout the nucleus (Supplemental eFigure 2A-2C). When one probe, usually the p-arm probe, is a quarter to half as frequent as the q-arm probe we consider this to be presumptive evidence for heterozygous deletion of the less frequent locus (Supplemental eFigure 2D-2F).

Finally, for additional support of this interpretation, we confirm that the opposite possibility is not supported by the FISH assay. That is, there is no evidence for "co-amplification" of *HER2* with the more frequent, usually q-arm probe, by demonstration that *HER2* and the more frequent alternative control signals, such as *RARA*, are NOT co-localized within tumor cell nuclei to the same limited geographic area of nuclei, as would be expected for two genes contained within the same amplicon. In such cancers, we assess the less frequent marker probe as showing heterozygous deletion (Supplemental eFigure 2). These criteria were used to interpret the alternative control probe status reported in Supplemental eTable 1. This strategy is similar to the strategy used to assess 1p and 19q deletions in the evaluation of central nervous system gliomas, particularly oligodendrogliomas, for 1p/19q co-deletion. This assessment is made through a comparison of chromosome 1 p-arm with chromosome 1 q-arm probes (1p36 / 1q25) to assess relative frequency of signals corresponding to each arm[21]. Similarly, a comparison of chromosome 19 q-arm with chromosome 19 p-arm probes (19q13 / 19p13) provides an assessment of relative frequency and, therefore, relative loss or reduction in one arm relative to the other as summarized in Table 2 for the *HER2* alternative control probe pairs [21].

HER2 Protein Expression by Immunohistochemistry. The HercepTest (Dako) as well as a laboratory-developed HER2 10H8-IHC assay were used to evaluate HER2 protein expression[5, 8, 13, 14] in tissue sections of breast cancers from the BCIRG-005 trial. In these "*HER2*-equivocal" breast cancers, 42 had available results from both IHC assays, 39 had results from only the 10H8-IHC assay, and 19 did not have any IHC available. Among "*HER2*-negative" breast cancers used in this study, all 100 breast cancers had IHC assay results available, 95 with both assays. When both IHC assays were available the Dako HercepTest was used for the analyses.

Statistical Methods. Hazard ratios (HRs) were estimated by using Cox proportional hazards regression models (Supplemental eTable 3).

eDiscussion

Since the 1980s we and others have been using various chromosome 17 markers to "normalize" the *HER2* gene copy number to determine if *HER2* is sufficiently increased to be considered "amplified"[1-3]. A ratio greater than or equal to 2.0, established for Southern hybridization in the 1980s, has proven to be a reasonable "cut-off" for separation of *HER2*-not-amplified from *HER2*-amplified breast cancers with the provision, described above, that utilization of heterozygous deleted control genes, such as *TP53* as described by Clark and McGuire[4], or the use of co-amplified genes, such as *TOP2A*[24], will lead to false-positive assessments in the former situation and false-negatives in the latter.

Although selection of appropriate chromosome 17 controls is important, the same control may not be useful for assessment of every breast cancer. For example, the use of chromosome 17 centromere (CEP17) for assessment of *HER2* by FISH has proven to be a useful control in most cancers. However, in breast cancers where the *HER2* amplicon is sufficiently large and extends in a centromeric direction with inclusion of alpha-satellite DNA adjacent to the centromere, the *HER2* gene copy number and the CEP17 copy number are both greatly increased (co-amplified), leading to a *HER2*-to-CEP17 ratio <2.0. In such cases, the use of an alternative control gene, such as *RARA*, provides a ratio substantially in excess of 2.0. In these HER2 ASCO-CAP FISH group 3A[8, 13] breast cancers an alternative control probe is very useful, as we describe and illustrate elsewhere (see figure 3 and figure 3 legend, in *Archives of Pathology and Laboratory Medicine* [13]).

© 2018 Press MF, et al. *JAMA Oncology.*

Supplemental Tables.

eTable 1. Assessment of Heterozygous Deletions by FISH using Pairwise Comparisons of Alternative Control Genes and Frequency of resulting *HER2* FISH ratios greater than 2.0 using these same Alternative Control Genes: ASCO-CAP Group 4 (*HER2*-Equivocal) and ASCO-CAP Group 5 (*HER2*-not-amplified) Breast Cancers.

	Heterozygous deletions by comparison of p-arm and q-arm probes						
ASCO-CAP FISH Group	SMS	*RARA*	*TP53*	*TOP2A*	D17S122	*HER2*	Total
Group 4	65 (65%)	0 (0%)	43 (43%)	8 (8%)	46 (46%)	0 (0%)	100
	Number with HER2-to-alternative control probe ratios ≥2.0						
ASCO-CAP FISH Group	SMS	*RARA*	*TP53*	*TOP2A*	D17S122	*HER2*	
Group 4	61 (61%)	7 (7%)	65 (65%)	25 (25%)	30 (30%)	NA	100
	Heterozygous deletions by comparison of p-arm and q-arm probes						
ASCO-CAP FISH Group	SMS	*RARA*	*TP53*	*TOP2A*	D17S122	*HER2*	
Group 5	30 (30%)	1 (1%)	0	3 (3%)	35 (35%)	0	100
	Number with HER2-to-alternative control probe ratios ≥2.0						
ASCO-CAP FISH Group	SMS	*RARA*	*TP53*	*TOP2A*	D17S122	*HER2*	
Group 5	37 (37%)	12 (12%)	2 (2%)	1 (1%)	11 (11%)	0	100

© 2018 Press MF, et al. *JAMA Oncology.*

eTable 2. Correlation of HER2 Protein Status by IHC among BCIRG-005 Trial Breast Cancers determined to be "*HER2*-Positive" using Various Alternative Control Probes (SMS, D17S122 and *TP53*) among ASCO-CAP Group 4 (*HER2* Equivocal) and ASCO-CAP Group 5 (*HER2*-not-amplified) Breast Cancers.

	IHC Status of ASCO-CAP FISH Group 4 Breast Cancers					
	IHC 0	IHC 1+	IHC 2+	IHC 3+	Total	None
All ASCO/CAP FISH Group 4	59 (73%)	18 (22%)	4 (5%)	0 (0%)	81	
Positive by HER2 / SMS Ratio ≥2.0	34 (71%)	10 (21%)	4 (8%)	0 (0%)	48	13
Negative by HER2 / SMS Ratio <2.0	25 (76%)	8 (24%)	0 (0%)	0 (0%)	33	6
Positive by HER2 / D17S122 Ratio ≥2.0	16 (73%)	4 (18%)	2 (9%)	0 (0%)	22	8
Negative by HER2 / D17S122 Ratio <2.0	43 (73%)	14 (24%)	2 (3%)	0 (0%)	59	11
Positive by HER2 / *TP53* Ratio ≥2.0	39 (78%)	8 (16%)	3 (6%)	0 (0%)	50	15
Negative by HER2 / *TP53* Ratio <2.0	20 (65%)	10 (32%)	1 (3%)	0 (0%)	31	4
	IHC Status of ASCO-CAP FISH Group 5 Breast Cancers					
All ASCO/CAP FISH Group 5	82 (82%)	17 (17%)	1 (1%)	0 (0%)	100	
Positive by HER2 / SMS Ratio ≥2.0	30 (81%)	7 (19%)	0 (0%)	0 (0%)	37 (100%)	
Negative by HER2 / SMS Ratio <2.0	52 (83%)	10 (16%)	1 (2%)	0 (0%)	63 (100%)	
Positive by HER2 / D17S122 Ratio ≥2.0	11 (100%)	0 (0%)	0 (0%)	0 (0%)	11 (100%)	
Negative by HER2 / D17S122 Ratio <2.0	71 (80%)	17 (19%)	1 (1%)	0 (0%)	89 (100%)	
Positive by HER2 / *TP53* Ratio ≥2.0	1 (50%)	1 (50%)	0 (0%)	0 (0%)	2 (100%)	
Negative by HER2 / *TP53* Ratio <2.0	81 (83%)	16 (16%)	1 (1%)	0 (0%)	98 (100%)	
Positive by HER2 / *RARA* Ratio ≥2.0	12 (100%)	0 (0%)	0 (0%)	0 (0%)	12 (100%)	
Negative by HER2 / *RARA* Ratio <2.0	70 (80%)	17 (19%)	1 (1%)	0 (0%)	88 (100%)	

© 2018 Press MF, et al. *JAMA Oncology.*

eTable 3. Outcomes for BCIRG-005 Trial Patients whose Breast Cancers were "*HER2*-Positive" using Various Alternative Control Probes (SMS, D17S122 and *TP53*) among ASCO-CAP Group 4 (*HER2*-Equivocal) and ASCO-CAP Group 5 (*HER2*-not-amplified) Breast Cancers.

	ASCO-CAP FISH Group 4 Breast Cancer Patients				
	Number of Subjects	DFS (number of events)	OS (number of events)	DFS, HR (95% CI) and P-values for logrank test	OS, HR (95% CI) and P-values for logrank test
Positive by *HER2* / SMS Ratio ≥2.0	61	25	13	1.0 (reference)	1.0 (reference)
Negative by *HER2* / SMS Ratio <2.0	39	11	9	0.73 (0.36 – 1.49) p=0.39	1.16 (0.50-2.72) p=0.73
Positive by *HER2* / D17S122 Ratio ≥2.0	30	12	7	1.0 (reference)	1.0 (reference)
Negative by *HER2* / D17S122 Ratio <2.0	70	24	15	0.81 (0.41-1.62) p=0.56	0.85 (0.35-2.08) p=0.72
Positive by *HER2* / *TP53*- Ratio ≥2.0	65	26	17	1.0 (reference)	1.0 (reference)
Negative by *HER2* / *TP53*- Ratio <2.0	35	10	5	0.66 (0.32 – 1.38) p=0.27	0.50 (0.18-1.35) p=0.16
	ASCO-CAP FISH Group 5 Breast Cancer Patients				
	Number of Subjects	DFS (number of events)	OS (number of events)	DFS, HR (95% CI) and P-values for logrank test	OS, HR (95% CI) and P-values for logrank test
Positive by *HER2* / SMS Ratio ≥2.0	37	14	10	1.0 (reference)	1.0 (reference)
Negative by HER2 / SMS Ratio <2.0	63	19	10	0.82 (0.41-1.64) p=0.58	0.59 (0.25-1.42) p=0.23
Positive by *HER2* / D17S122 Ratio ≥2.0	11	5	3	1.0 (reference)	1.0 (reference)
Negative by HER2 / D17S122 Ratio <2.0	89	28	17	0.60 (0.23-1.55) p=0.29	0.54 (0.16-1.86) p=0.32
Positive by *HER2* / *TP53*- Ratio ≥2.0	2	1	0	1.0 (reference)	1.0 (reference)
Negative by *HER2* / *TP53*- Ratio <2.0	98	32	20	0.94 (0.13-6.93) p=0.96	na

© 2018 Press MF, et al. *JAMA Oncology.*

Supplemental Figures.

eFigure 1. Relative copy number of *HER2 / ERBB2* and Genomic Sites used as Alternate Controls (*LIS1*, *TP53*, D17S122, *RAI1* SMS, *RARA-TOP2A*) for Assessment of HER2 Status by FISH (METABRIC COHORT. SNP chip data; N = 1980).

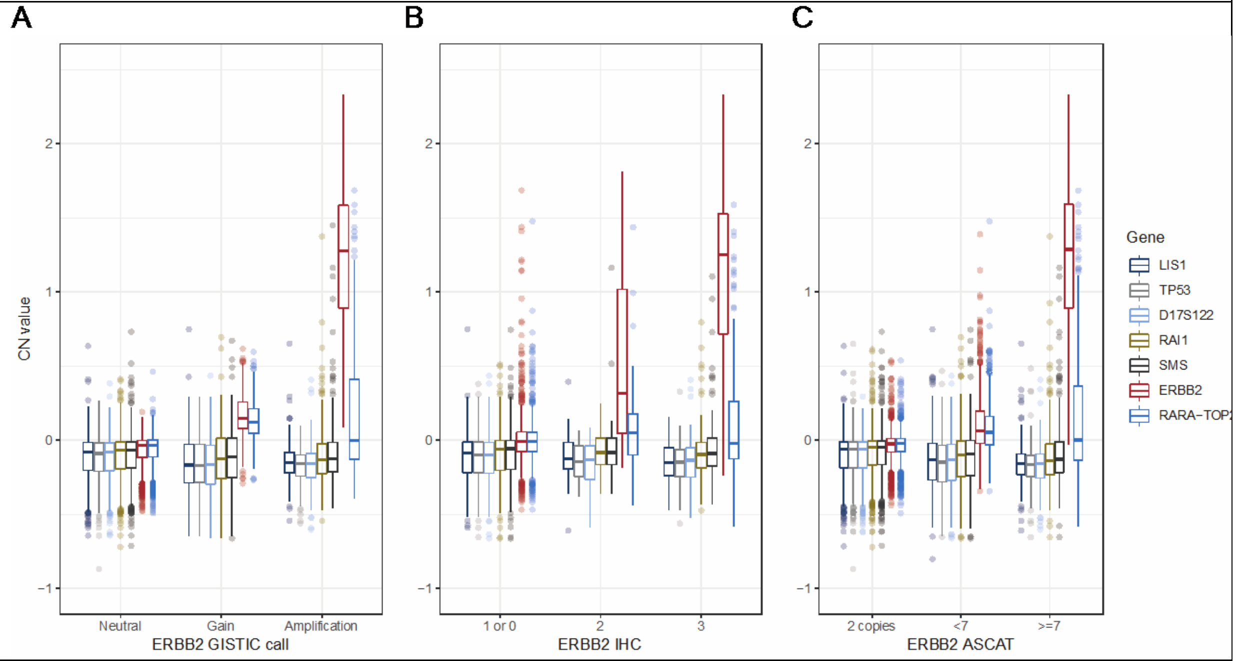

A.) Boxplot for alternative genomic site CN probes based on GISTIC (Genomic Identification of Significant Targets in Cancer) copy number calls. B.) Boxplot for alternative genomic site CN probes based on HER2 IHC (immunohistochemistry) values. C.) Boxplot for alternative genomic site CN probes based on HER2 ASCAT (allele-specific copy number analysis of tumors) calls.

© 2018 Press MF, et al. *JAMA Oncology.*

eFigure 2. Assessment of Heterozygous Deletion Among Chromosome 17 genomic sites by comparison of p-arm (*TP53*, SMS, D17S122) with q-arm (*RARA*, *TOP2A*, *HER2*) probes.

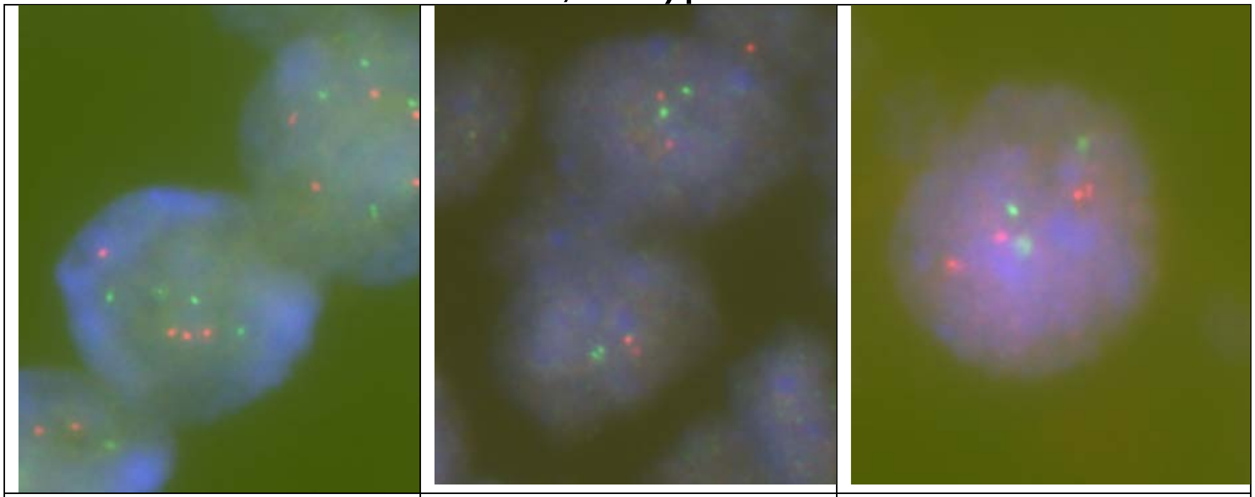

A. *TOP2A* (orange), *TP53* (green) | B. SMS (orange), *RARA* (green) | C. *HER2* (orange), D17S122 (green)

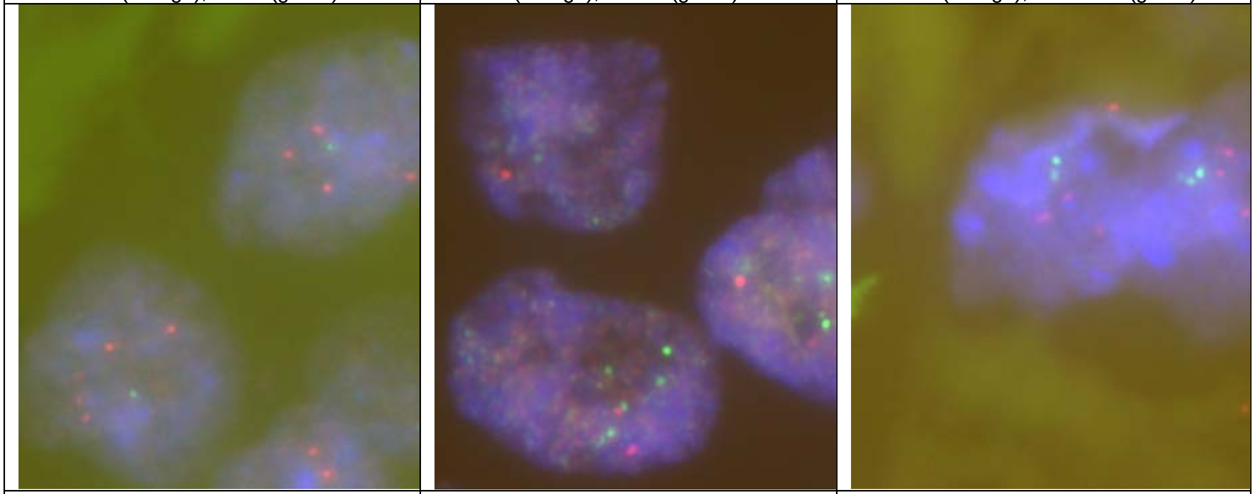

D. *TOP2A* (orange), *TP53* (green) | E. SMS (orange), *RARA* (green) | F. *HER2*(orange), D17S122(green)

Breast cancers with similar numbers of p-arm and q-arm markers are interpreted as showing a lack of deletion at those specific genomic sites (A – C). A.) For example, pairwise comparison of *TP53* (green) with *TOP2A* (red) shows similar gene copy numbers with an average *TP53* gene copy per tumor cell of 4.05 and an average *TOP2A* of 3.95 copies per tumor cell for a *TP53* / *TOP2A* ratio of 1.03. Therefore, this pairwise comparison was interpreted as "no deletion" at either site. BCIRG 01502_(Dapi+Orange+Green). B.) Pairwise comparison of SMS (red) with *RARA* (green) shows similar gene copy number with SMS and *RARA* both having an average copy number of 1.90 per tumor cell nucleus for a SMS / *RARA* ratio of 1.00. This pairwise comparison was interpreted as "no deletion" at either site. BCIRG array 1 A8d_(Dapi+Orange+Green) C.) Pairwise comparison of D17S122 (green) with *HER2* (red) also shows similar gene copy numbers with D17S122 averages of 2.35 copies per tumor cell and *HER2* copy numbers of 2.90 per tumor cell for a *HER2* / D17S122 ratio of 1.23 or, as in Table 2, D17S122 / *HER2* ratio of 0.81. This pairwise comparison in the carcinoma cells was interpreted as "no deletion" in either site. BCIRGB IV B5q_(Dapi+Orange+Green) **(D.-F.) In contrast, an at least 25% reduction of one of these sites relative to the other site is interpreted as "heterozygous deletion", pending confirmation based on additional considerations of signal groupings (see Table 2)**. D.) Heterozygous deletion of *TP53.* The average *TP53* copy number for this case was 1.50, while the average *TOP2A* copy number was 2.80 for a *TP53* / *TOP2A* ratio of 0.54, consistent with heterozygous deletion of *TP53* relative to *TOP2A*. BCIRG Array 1 B 5m (Dapi+Orange+Green) E.) Heterozygous deletion of SMS. The average SMS copy number for this case was 1.65, while the average *RARA* copy number was 3.60 for a SMS / *RARA* ratio of 0.46, consistent with heterozygous deletion of SMS relative to *RARA*. BCIRG Array 1 C4h (Dapi+Orange+Green) F.) Heterozygous deletion of D17S122. The average D17S122 copy number for this case was 2.00, while the average *HER2* copy number was 3.05 for a D17S122 / *HER2* ratio of 0.66, consistent with heterozygous deletion of D17S122 relative to *HER2*. BCIRG00014 ARRAY 1 A1n_(Dapi+Orange+Green). Original magnification of all images: 1000x.

© 2018 Press MF, et al. *JAMA Oncology.*

eFigure 3. Comparison of Clinical Outcomes for ASCO-CAP Group 4 (*HER2*-Equivocal) and ASCO-CAP Group 5 (*HER2*-not-amplified) Breast Cancer Patients. Kaplan-Meier plots.

A. Disease-Free Survival of ASCO-CAP FISH Group 4 (*HER2*-Equivocal) Compared to ASCO-CAP FISH Group 5 (*HER2*-negative)

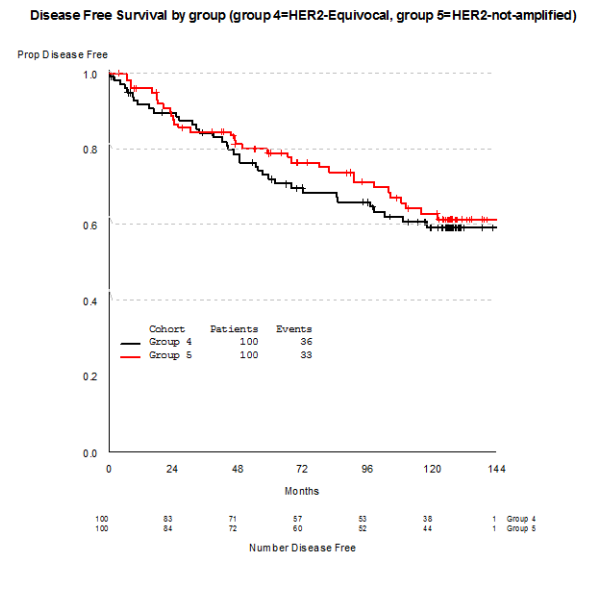

B. Overall Survival of ASCO-CAP FISH Group 4 (*HER2*-Equivocal) Compared to ASCO-CAP FISH Group 5 (*HER2*-negative)

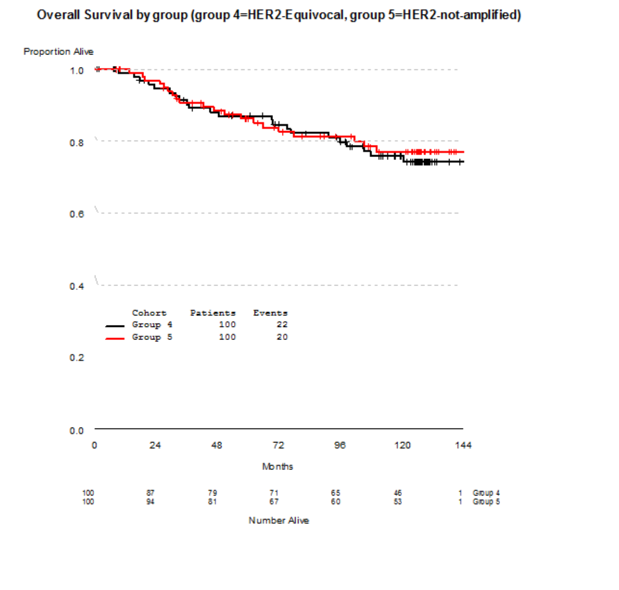

C. ASCO-CAP FISH Group 4 (*HER2*-Equivocal): OS for Alternative Control Probe D17S122 (*HER2* / D17S122) Ratios >2.0 versus <2.0.

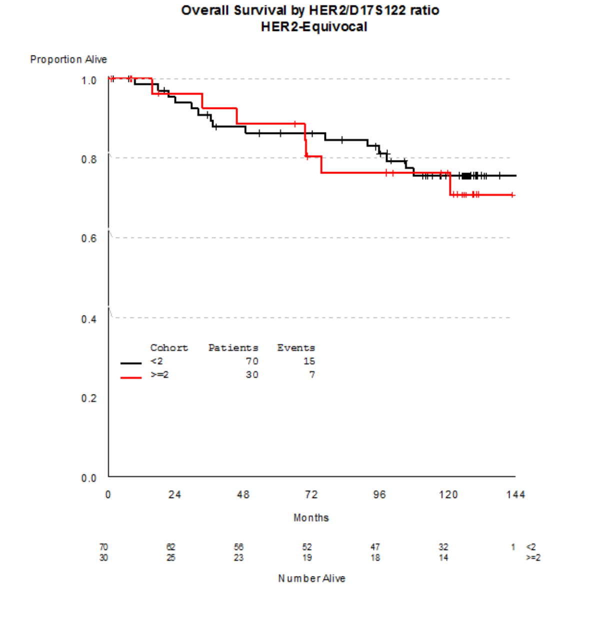

D. ASCO-CAP FISH Group 5 (*HER2*-negative): OS for Alternative Control Probe D17S122 (*HER2* / D17S122) Ratios >2.0 versus <2.0.

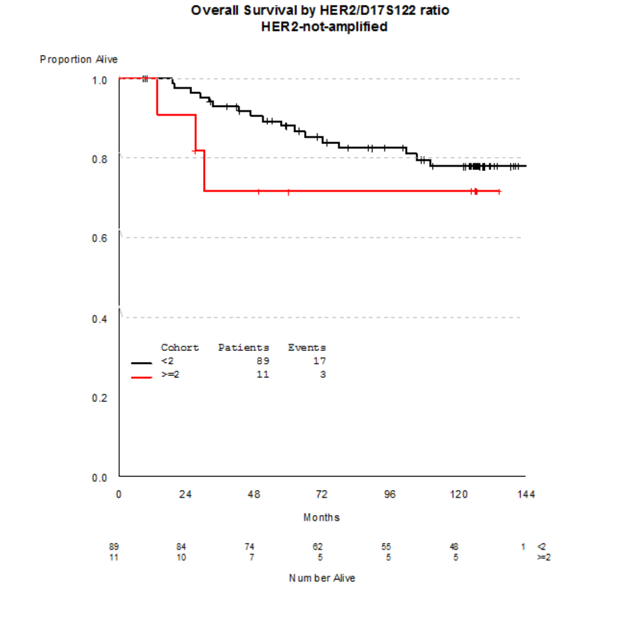

E. ASCO-CAP FISH Group 4 (*HER2*-Equivocal): OS for Alternative Control Probe SMS (*HER2* / SMS) Ratios >2.0 versus <2.0.

F. ASCO-CAP FISH Group 5 (*HER2*-negative): OS for Alternative Control Probe SMS (*HER2* / SMS) Ratios >2.0 versus <2.0.

© 2018 Press MF, et al. *JAMA Oncology.*

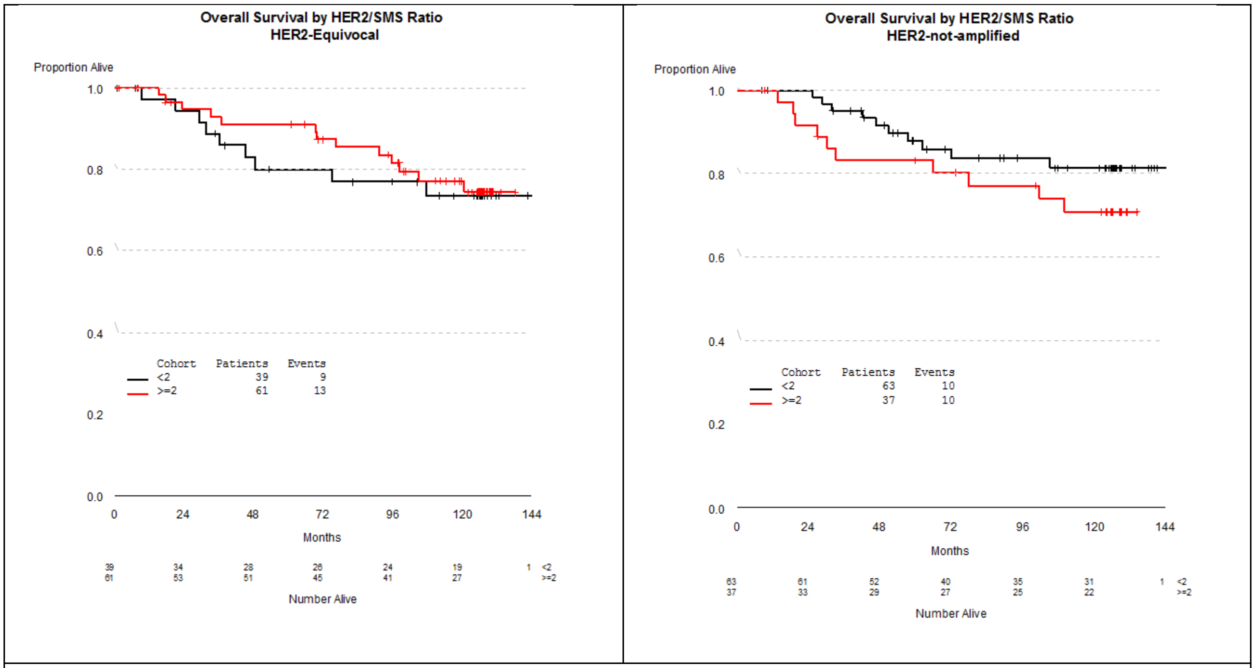

(A.) Disease-Free Survival of ASCO-CAP FISH Group 4 (*HER2*-Equivocal) Compared to ASCO-CAP FISH Group 5 (*HER2*-not-amplified). There is no significant difference in disease-free survival for the 100 patients with ASCO-CAP FISH Group 4 (*HER2*-Equivocal) breast cancers compared to the 100 patients with ASCO-CAP FISH Group 5 (*HER2*-negative) breast cancers. (B.) Overall Survival of ASCO-CAP FISH Group 4 (*HER2*-Equivocal) Compared to ASCO-CAP FISH Group 5 (*HER2*-negative) breast cancer patients. There is no significant difference in overall survival for the 100 patients with ASCO-CAP FISH Group 4 (*HER2*-Equivocal) breast cancers compared to the 100 patients with ASCO-CAP FISH Group 5 (*HER2*-negative) breast cancers. (C.) ASCO-CAP FISH Group 4 (*HER2*-Equivocal): OS for Alternative Control Probe D17S122 (*HER2* / D17S122) Ratios $\geq$2.0 versus <2.0. Among women with "*HER2*-equivocal" breast cancers with an D17S122 alternative control probe ratio $\geq$2.0 does not identify a subgroup with a worse overall survival. (D.) ASCO-CAP FISH Group 5 (*HER2*-negative): OS for Alternative Control Probe D17S122 (*HER2* / D17S122). Interestingly, among women with *HER2*-negative (*HER2*-not-amplified) breast cancers those with an D17S122 alternative control probe ratio $\geq$2.0 appears to identify a subgroup with a numerically slightly worse overall survival which is not statistically significant. (E.) ASCO-CAP FISH Group 4 (*HER2*-Equivocal): OS for Alternative Control Probe SMS (*HER2* / SMS) Ratios $\geq$2.0 versus <2.0. Among women with "*HER2*-equivocal" breast cancers, those who had a SMS alternative control probe ratio $\geq$2.0 appear to have a slightly better overall survival than those whose breast cancers had a SMS alternative control probe ratio <2.0; however, this difference was not significant. (F.) ASCO-CAP FISH Group 5 (*HER2*-negative): OS for Alternative Control Probe SMS (*HER2* / SMS) Ratios $\geq$2.0 versus <2.0. The differences in OS among ASCO-CAP group 5 breast cancer patients with alternative control probe SMS (*HER2* / SMS) ratios $\geq$2.0 versus <2.0 were not statistically significant.

© 2018 Press MF, et al. *JAMA Oncology.*

eReferences

1. Slamon DJ, Clark GM. Amplification of c-erbB-2 and aggressive human breast tumors? *Science.* 1988; 240(4860):1795-1798.
2. Slamon DJ, Clark GM, Wong SG, Levin WJ, Ullrich A, McGuire WL. Human breast cancer: correlation of relapse and survival with amplification of the HER-2/neu oncogene. *Science.* 1987; 235(4785):177-182.
3. Slamon DJ, Godolphin W, Jones LA, Holt JA, Wong SG, Keith DE, Levin WJ, Stuart SG, Udove J, Ullrich A, Press MF. Studies of the HER-2/neu proto-oncogene in human breast and ovarian cancer. *Science.* 1989; 244(4905):707-712.
4. Clark GM, McGuire WL. Follow-up study of HER-2/neu amplification in primary breast cancer. *Cancer Res.* 1991; 51(3):944-948.
5. Press MF, Sauter G, Bernstein L, Villalobos IE, Mirlacher M, Zhou JY, Wardeh R, Li YT, Guzman R, Ma Y, Sullivan-Halley J, Santiago A, Park JM, Riva A, Slamon DJ. Diagnostic evaluation of HER-2 as a molecular target: an assessment of accuracy and reproducibility of laboratory testing in large, prospective, randomized clinical trials. *Clin Cancer Res.* 2005; 11(18):6598-6607.
6. Eiermann W, Pienkowski T, Crown J, Sadeghi S, Martin M, Chan A, Saleh M, Sehdev S, Provencher L, Semiglazov V, Press M, Sauter G, Lindsay MA, Riva A, Buyse M, Drevot P, Taupin H, Mackey JR. Phase III study of doxorubicin/cyclophosphamide with concomitant versus sequential docetaxel as adjuvant treatment in patients with human epidermal growth factor receptor 2-normal, node-positive breast cancer: BCIRG-005 trial. *J Clin Oncol.* 2011; 29(29):3877-3884.
7. Mackey JR, Pienkowski T, Crown J, Sadeghi S, Martin M, Chan A, Saleh M, Sehdev S, Provencher L, Semiglazov V, Press MF, Sauter G, Lindsay M, Houe V, Buyse M, Drevot P, Hitier S, Bensfia S, Eiermann W, Translational Research In Oncology / Breast Cancer International Research Group i. Long-term outcomes after adjuvant treatment of sequential versus combination docetaxel with doxorubicin and cyclophosphamide in node-positive breast cancer: BCIRG-005 randomized trial. *Ann Oncol.* 2016; 27(6):1041-1047.
8. Press MF, Sauter G, Buyse M, Fourmanoir H, Quinaux E, Tsao-Wei DD, Eiermann W, Robert N, Pienkowski T, Crown J, Martin M, Valero V, Mackey JR, Bee V, Ma Y, Villalobos I, Campeau A, Mirlacher M, Lindsay MA, Slamon DJ. HER2 Gene Amplification Testing by Fluorescent In Situ Hybridization (FISH): Comparison of the ASCO-College of American Pathologists Guidelines With FISH Scores Used for Enrollment in Breast Cancer International Research Group Clinical Trials. *J Clin Oncol.* 2016; 34 (29):3518-3528.
9. Slamon D, Eiermann W, Robert N, Pienkowski T, Martin M, Press M, Mackey J, Glaspy J, Chan A, Pawlicki M, Pinter T, Valero V, Liu MC, Sauter G, von Minckwitz G, Visco F, Bee V, Buyse M, Bendahmane B, Tabah-Fisch I, Lindsay MA, Riva A, Crown J, Breast Cancer International Research G. Adjuvant trastuzumab in HER2-positive breast cancer. *N Engl J Med.* 2011; 365(14):1273-1283.
10. Valero V, Forbes J, Pegram MD, Pienkowski T, Eiermann W, von Minckwitz G, Roche H, Martin M, Crown J, Mackey JR, Fumoleau P, Rolski J, Mrsic-Krmpotic Z, Jagiello-Gruszfeld A, Riva A, Buyse M, Taupin H, Sauter G, Press MF, Slamon DJ. Multicenter phase III randomized trial comparing docetaxel and trastuzumab with docetaxel, carboplatin, and trastuzumab as first-line chemotherapy for patients with HER2-gene-amplified metastatic breast cancer (BCIRG 007 study): two highly active therapeutic regimens. *J Clin Oncol.* 2011; 29(2):149-156.
11. Mass RD, Press MF, Anderson S, Cobleigh MA, Vogel CL, Dybdal N, Leiberman G, Slamon DJ. Evaluation of clinical outcomes according to HER2 detection by fluorescence in situ hybridization in women with metastatic breast cancer treated with trastuzumab. *Clin Breast Cancer.* 2005; 6(3):240-246.
12. Press MF, Bernstein L, Thomas PA, Meisner LF, Zhou JY, Ma Y, Hung G, Robinson RA, Harris C, El-Naggar A, Slamon DJ, Phillips RN, Ross JS, Wolman SR, Flom KJ. HER-2/neu gene amplification characterized by fluorescence in situ hybridization: poor prognosis in node-negative breast carcinomas. *J Clin Oncol.* 1997; 15(8):2894-2904.
13. Press MF, Villalobos I, Santiago A, Guzman R, Cervantes M, Gasparyan A, Campeau A, Ma Y, Tsao-Wei DD, Groshen S. Assessing the New American Society of Clinical Oncology/College of American Pathologists Guidelines for HER2 Testing by Fluorescence In Situ Hybridization: Experience of an Academic Consultation Practice. *Arch Pathol Lab Med.* 2016; 140(11):1250-1258.
14. Press MF, Slamon DJ, Flom KJ, Park J, Zhou JY, Bernstein L. Evaluation of HER-2/neu gene amplification and overexpression: comparison of frequently used assay methods in a molecularly characterized cohort of breast cancer specimens. *J Clin Oncol.* 2002; 20(14):3095-3105.

© 2018 Press MF, et al. *JAMA Oncology.*

15. Wolff AC, Hammond ME, Hicks DG, Dowsett M, McShane LM, Allison KH, Allred DC, Bartlett JM, Bilous M, Fitzgibbons P, Hanna W, Jenkins RB, Mangu PB, Paik S, Perez EA, Press MF, Spears PA, Vance GH, Viale G, Hayes DF, American Society of Clinical O, College of American P. Recommendations for human epidermal growth factor receptor 2 testing in breast cancer: American Society of Clinical Oncology/College of American Pathologists clinical practice guideline update. *J Clin Oncol.* 2013; 31(31):3997-4013.

16. Wolff AC, Hammond ME, Hicks DG, Dowsett M, McShane LM, Allison KH, Allred DC, Bartlett JM, Bilous M, Fitzgibbons P, Hanna W, Jenkins RB, Mangu PB, Paik S, Perez EA, Press MF, Spears PA, Vance GH, Viale G, Hayes DF, American Society of Clinical O, College of American P. Recommendations for human epidermal growth factor receptor 2 testing in breast cancer: American Society of Clinical Oncology/College of American Pathologists clinical practice guideline update. *Arch Pathol Lab Med.* 2014; 138(2):241-256.

17. Donaldson AR, Shetty S, Wang Z, Rivera CL, Portier BP, Budd GT, Downs-Kelly E, Lanigan CP, Calhoun BC. Impact of an alternative chromosome 17 probe and the 2013 American Society of Clinical Oncology and College of American Pathologists guidelines on fluorescence in situ hybridization for the determination of HER2 gene amplification in breast cancer. *Cancer.* 2017; 123(12):2230-2239.

18. Shah MV, Wiktor AE, Meyer RG, Tenner KS, Ballman KV, Green SJ, Sukov WR, Ketterling RP, Perez EA, Jenkins RB. Change in Pattern of HER2 Fluorescent in Situ Hybridization (FISH) Results in Breast Cancers Submitted for FISH Testing: Experience of a Reference Laboratory Using US Food and Drug Administration Criteria and American Society of Clinical Oncology and College of American Pathologists Guidelines. *J Clin Oncol.* 2016; 34(29):3502-3510.

19. Sneige N, Hess KR, Multani AS, Gong Y, Ibrahim NK. Prognostic significance of equivocal human epidermal growth factor receptor 2 results and clinical utility of alternative chromosome 17 genes in patients with invasive breast cancer: A cohort study. *Cancer.* 2017; 123(7):1115-1123.

20. Agersborg S, Mixon C, Nguyen T, Aithal S, Sudarsanam S, Blocker F, Weiss L, Gasparini R, Jiang S, Chen W, Hess G, Albitar M. Immunohistochemistry and alternative FISH testing in breast cancer with HER2 equivocal amplification. *Breast Cancer Research and Treatment* 2018; 170:321–328.

21. Smith JS, Perry A, Borell TJ, Lee HK, O'Fallon J, Hosek SM, Kimmel D, Yates A, Burger PC, Scheithauer BW, Jenkins RB. Alterations of chromosome arms 1p and 19q as predictors of survival in oligodendrogliomas, astrocytomas, and mixed oligoastrocytomas *Journal of Clinical Oncology* 2000; 18 636-645

22. Korshunov A, Sycheva R, Golanov A. Molecular stratification of diagnostically challenging high-grade gliomas composed of small cells: the utility of fluorescence in situ hybridization. *Clin Cancer Res.* 2004; 10(23):7820-7826.

23. Bacus SS, Bacus JW, Slamon DJ, Press MF. HER-2/neu oncogene expression and DNA ploidy analysis in breast cancer. *Arch Pathol Lab Med.* 1990; 114(2):164-169.

24. Press MF, Sauter G, Buyse M, Bernstein L, Guzman R, Santiago A, Villalobos IE, Eiermann W, Pienkowski T, Martin M, Robert N, Crown J, Bee V, Taupin H, Flom KJ, Tabah-Fisch I, Pauletti G, Lindsay MA, Riva A, Slamon DJ. Alteration of topoisomerase II-alpha gene in human breast cancer: association with responsiveness to anthracycline-based chemotherapy. *J Clin Oncol.* 2011; 29(7):859-867.

© 2018 Press MF, et al. *JAMA Oncology.*
